# P20 Persistent anti-PR3 ANCA positivity in patient with extrapulmonary tuberculosis

**DOI:** 10.1093/rap/rkae117.051

**Published:** 2024-11-05

**Authors:** Thushyanthan Guruparan, Tanjina Nasrin, Pratiksha Karki, Riotsuko Saito, Zehra Gul, Leslie Goh, Gurdeep Dulay

**Affiliations:** Queen Alexandra Hospital, Portsmouth, United Kingdom; Queen Alexandra Hospital, Portsmouth, United Kingdom; Queen Alexandra Hospital, Portsmouth, United Kingdom; Queen Alexandra Hospital, Portsmouth, United Kingdom; Queen Alexandra Hospital, Portsmouth, United Kingdom; Queen Alexandra Hospital, Portsmouth, United Kingdom; Queen Alexandra Hospital, Portsmouth, United Kingdom

## Abstract

**Introduction:**

Granulomatosis with polyangiitis (GPA) and tuberculosis (TB) are both granulomatous disease which can have similar clinical manifestations and organ involvement. This report discusses a case of such overlapping clinical presentation and anti-neutrophil cytoplasmic antibodies (ANCA) positivity, leading to diagnostic uncertainty.

**Case description:**

A 40-year-old female who lived in sub-Saharan Africa until two years ago presented with a two month history of general malaise, significant weight loss, severe anorexia, diarrhoea, vomiting and nausea. She had no night sweats, blood in stool, abdominal pain, respiratory or cardiac symptoms, sino-nasal symptoms, rashes or neurological features. On examination, she appeared severely malnourished. Blood tests revealed microcytic anaemia (Hb 83 g/l, MCV 69fl), thrombocytosis, hypoalbuminemia (15 g/l), CRP 26 mg/l, elevated procalcitonin (1.46 ug/l), elevated LDH 263U/l. She had a positive double-stranded DNA and anti-cardiolipin IgM. ANCA was moderately positive with an anti-PR3 titre of 78 U/ml. Computerised tomography (CT) chest to pelvis revealed widespread necrotic lymphadenopathy, bilateral pulmonary emboli with infarcts, pancolitis and non-specific gall bladder thickening. Endoscopy revealed inflamed featureless colon with no microscopic granulomata. Acid-fast bacilli were isolated on endobronchial ultrasound guided biopsy of the necrotic nodes leading to confirmation of the diagnosis of extrapulmonary disseminated tuberculosis. Clinical improvement and improvement in CRP and anti-PR3 titre (48 U/ml) were seen following two weeks of anti-tuberculous treatment and a weaning course of steroids for colitis.

**Discussion:**

Often GPA and tuberculosis can have similar presentations including lung and gastrointestinal involvement along with lymphadenopathy. This case demonstrates how ANCA and PR3 positivity can also be a common feature in these two conditions. Correct diagnosis is crucial in preventing harm to the patient as a diagnosis of GPA would require prompt and aggressive immunosuppression which may result in disseminated mycobacterial infection in the case of misdiagnosis; similarly, anti-mycobacterial therapy is unlikely control systemic GPA.

In our patient, although the enteritis, general malaise and weight loss can be features of vasculitis and tuberculosis, careful history suggested no convincing other features of GPA, including the absence of ear, nose, throat, skin, neurological or renal involvement. The social history yielded migration from sub-Saharan Africa where tuberculosis is much more prevalent. A useful distinguishing biochemical test was the elevated procalcitonin which points towards a bacterial infection rather than an autoimmune process. Ultimate conclusive evidence of mycobacterial infection requires either positive acid-fast bacilli on staining or culture; lymph node biopsy allowed us to promptly and correctly diagnose this patient in the absence of sputum or pulmonary tuberculosis. Initiation of anti-tuberculous treatment rapidly settled this patient’s symptoms with a falling CRP.

This case report highlights the fact that tuberculosis can mimic ANCA-associated vasculitis with positive anti-PR3 antibodies. In such cases, careful history taking, appropriate imaging and positive acid fast bacilli on staining or culture from sputum or tissue are important for prompt diagnosis and treatment. Both disseminated tuberculosis and systemic GPA can be rapidly progressive and severe if untreated. Although literature described the co-existence of both conditions, this is very rare. Correct diagnosis is key in initiation of the widely different treatment strategies, with the treatment for GPA potentially causing harm in disseminated tuberculosis.

**Key learning points:**

• ANCA positivity in tuberculosis has been documented in the literature and positive anti-PR3 cannot be used solely to diagnose GPA. Therefore, a thorough history, examination, imaging and tissue examination, including tuberculosis culture and acid-fast staining, are recommended.

